# Performance and impact of rapid multiplex PCR on diagnosis and treatment of ventilated hospital-acquired pneumonia in patients with extended-spectrum β-lactamase-producing *Enterobacterales* rectal carriage

**DOI:** 10.1186/s13613-024-01348-5

**Published:** 2024-07-29

**Authors:** Pierre Bay, Vincent Fihman, Paul-Louis Woerther, Bastien Peiffer, Ségolène Gendreau, Romain Arrestier, Pascale Labedade, Elsa Moncomble, Antoine Gaillet, Guillaume Carteaux, Nicolas de Prost, Armand Mekontso Dessap, Keyvan Razazi

**Affiliations:** 1 AP-HP (Assistance Publique-Hôpitaux de Paris), Hôpitaux Universitaires Henri Mondor, DMU Médecine, Service de Médecine Intensive Réanimation, CHU Henri Mondor, 51, Av. de Lattre de Tassigny, 94010 Créteil CEDEX, France; 2 Faculté de Santé de Créteil, UPEC (Université Paris Est Créteil), IMRB, GRC CARMAS, 94010 Créteil, France; 3 UPEC (Université Paris Est), INSERM, Unité U955, Équipe 18, 94010 Créteil, France; 4 Département de Virologie, Bactériologie, Parasitologie-Mycologie, AP-HP (Assistance Publique-Hôpitaux de Paris), Hôpitaux Universitaires Henri Mondor, 94010 Créteil, France; 5 UPEC (Université Paris Est), EA 7380 Dynamic, Ecole Nationale Vétérinaire d’Alfort, USC Anses, Créteil, France; 6 Assistance Publique-Hôpitaux de Paris AP-HP, Hôpital Henri Mondor, DMU Médecine, Créteil, France

**Keywords:** Ventilator-associated pneumonia, Multiplex PCR, Antimicrobial stewardship, ESBL, Nosocomial pneumonia, Carbapenem, Intensive care unit

## Abstract

**Background:**

Antimicrobial stewardship (AMS) for ventilator-associated pneumonia (VAP) or ventilated hospital-acquired pneumonia (vHAP) in extended-spectrum β-lactamase-producing *Enterobacterales* (ESBL-E) carriers is challenging. BioFire® FilmArray® Pneumonia plus Panel (mPCR) can detect bacteria and antibiotic resistance genes, including *bla*_CTX-M_, the most common ESBL-encoding gene.

**Methods:**

This monocentric, prospective study was conducted on a group of ESBL-E carriers from March 2020 to August 2022. The primary objective was to evaluate the concordance between the results of mPCR and conventional culture performed on respiratory samples of ESBL-E carriers to investigate suspected VAP/vHAP. The secondary objective was to appraise the impact of performing or not mPCR on initial antibiotic therapy adequacy in ESBL-E carriers with confirmed VAP/vHAP.

**Results:**

Over the study period, 294 patients with ESBL-E carriage were admitted to the ICU, of who 168 (57%) were mechanically ventilated. (i) Diagnostic performance of mPCR was evaluated in suspected 41 episodes of VAP/vHAP: *bla*_CTX-M_ gene was detected in 15/41 (37%) episodes, where 9/15 (60%) were confirmed ESBL-E-induced pneumonia. The culture and *bla*_CTX-M_ were concordant in 35/41 (85%) episodes, and in all episodes where *bla*_CTX-M_ was negative (n = 26), the culture never detected ESBL-E. (ii) The impact of mPCR on initial antibiotic therapy adequacy was assessed in 95 episodes of confirmed VAP/vHAP (22 episodes were tested with mPCR and 73 without); 47 (49%) episodes were ESBL-E-induced, and 24 (25%) were carbapenem-resistant bacteria-induced. The use of mPCR was significantly associated with higher prescription of adequate empirical antibiotic therapy in the multivariable logistic regression (adjusted odds ratio (aOR) (95% CI) of 7.5 (2.1–35.9), p = 0.004), propensity-weighting (aOR of 5.9 (1.6–22.1), p = 0.008), and matching-cohort models (aOR of 5.8 (1.5–22.1), p = 0.01).

**Conclusion:**

mPCR *bla*_CTX-M_ showed an excellent diagnostic value to rule out the diagnosis of ESBL-E related pneumonia in ESBL-E carriers with suspected VAP/vHAP. In addition, in patients with confirmed VAP/vHAP, a mPCR-based antibiotic therapy was associated with an increased prescription of adequate empirical antibiotic therapy. Performing mPCR on respiratory samples seems to be a promising tool in ESBL-E carriers with suspected vHAP/VAP. However, if mPCR is used in very low pre-test clinical probability of pneumonia, due to the high sensitivity and the rate of overdiagnosed pneumonia, the risk of overconsumption of carbapenem may prevail. Further studies are warranted.

**Supplementary Information:**

The online version contains supplementary material available at 10.1186/s13613-024-01348-5.

## Introduction

The most common indication for antibiotic treatment in the intensive care unit (ICU) that accounts for half of its prescriptions is suspected lower respiratory tract infection [1]. The need for early use of adequate antibiotic regimen in the ICU should be weighed against the risk of promoting multidrug-resistant (MDR) bacteria via unnecessary broad spectrum antibiotic therapy [2]. Antimicrobial stewardship (AMS) is even more challenging in patients whose digestive tracts are colonised with extended-spectrum β-lactamase-producing *Enterobacterales* (ESBL-E), a known risk factor for infections [2]. The French guidelines recommend the use of carbapenems for suspected ventilator-associated pneumonia (VAP) in ESBL-E colonised patients who are immunosuppressed or presenting signs of severity [3]. However, ESBL-E related VAP accounted for only 7% of infection-related ventilator-associated complications in ESBL-E carriers, making carbapenems prescription often unnecessary [4]. The lack of reliable predictor of ESBL-E-related pneumonia in ESBL-E carriers and the relatively high prevalence of pneumonia caused by carbapenem-resistant bacteria (CRB) in ESBL-E carriers are strong arguments to look for novel diagnostic approaches [4].

BioFire® FilmArray® Pneumonia plus Panel (bioMérieux, France) is a rapid multiplex PCR (mPCR) test that can detect in 1.5 h, when performed on respiratory samples, 18 bacteria, nine viruses, and seven antibiotic resistance genes, including *bla*_CTX-M_, the most widely represented ESBLs in *Enterobacterales* isolated in the USA and Europe today [5]. Despite its good diagnostic value [6–10], mPCR showed conflicting results on AMS [11, 12] and has never been tested in ESBL-E carriers, a specific population with high risk of ESBL-E-related infections.

The primary objective of this study was to evaluate the concordance between the results of mPCR and conventional culture applied on respiratory samples of ESBL-E carriers with suspected VAP/vHAP. The secondary objective was to appraise the impact of performing or not mPCR on initial antibiotic therapy adequacy in ESBL-E carriers with confirmed vHAP/VAP.

## Methods

### Setting and patients

This monocentric observational prospective study, was conducted from March 2020 to August 2022 in a medical ICU of a university hospital. We included all ESBL-E carriers receiving invasive mechanical ventilation for more than 2 days and those requiring invasive mechanical ventilation for hospital-acquired pneumonia (i.e., vHAP). Intestinal carriage of ESBL-E was screened by rectal swabbing at ICU admission and weekly afterwards. The following data were collected: age, sex, comorbidities, Simplifed Acute Physiology Score (SAPS II), main reason for admission, antibiotic class received during ICU stay, clinical and biological features at time of sampling, and empirical antibiotic class initiated after sampling, after mPCR results, after quantitative culture results and after antibiotic susceptibility testing (AST) results.

Pneumonia was clinically suspected upon discovering new or persistent pulmonary infiltrates on chest X-ray associated with two of the following: purulent respiratory secretions, fever or hypothermia (body temperature greater > 38 or < 36 °C, respectively), leukocytosis or leukopenia (white blood cells count ≥ 12 × 10^9^ or ≤ 4 × 10^9^/L, respectively) [4, 13]. Confirmed pneumonia was defined by quantitative culture from a protected telescopic catheter samples (≥ 10^3^ CFU/mL), bronchoalveolar lavage fluid (≥ 10^4^ CFU/mL), or endotracheal aspirate (≥ 10^5^ CFU/mL). These thresholds were not applied to mPCR results. Noteworthy, the BioFire Pneumonia test was not initially validated on protected telescopic catheter, but recent studies have evidenced its good diagnostic value on such samples [8, 9, 14, 15]. VAP was defined as pneumonia developing after ≥ 48 h of endotracheal intubation, whereas vHAP was defined as pneumonia occurring within the 24 h preceding intubation in patients hospitalised for at least 48 h [16].

### Microbiological analysis

Conventional microbiological analyses were conducted in compliance with EUCAST recommendations and included quantitative culture, bacterial identifications using Matrix-Assisted Laser Desorption/Ionisation-Time-Of-Flight mass spectrometer (Microflex LT, Bruker Daltonics, Bremen, Germany), and AST performed using disk diffusion method on Mueller–Hinton media (Bio-Rad, Marnes-la-Coquette, France) on colonies isolated after the primary culture. In *Enterobacterales*, ESBL were phenotypically detected on AST if a difference of more than 5 mm was observed between the discs “Cefepime” and “Cefepime + clavulanate” and/or using a double-disk synergy test [17]. A carbapenemase was phenotypically suspected on AST when the ertapenem diameter was below the susceptibility breakpoint and confirmed by qualitative lateral flow immunoassay (NG-Test® CARBA-5, NG-Biotech, Guipry, France). FilmArray® Pneumonia plus panel was implemented according to the manufacturer’s instructions using 200 µL of the mucolytic SL-diluted solution (Copan) as a sample for the pouch-based mPCR with FilmArray Torch instrument [18]. Intensivists obtained the results of mPCR 24/7 and within two hours from receiving the sample at the laboratory. mPCR was performed whatever direct smear examination results. For endotracheal aspirates, mPCR was performed only if there was polymorphonuclear cells without squamous epithelial cells.

### Diagnostic performance of mPCR *bla*_CTX-M_ in ESBL-E carriers with suspected of VAP/vHAP

The primary objective of the study was to evaluate prospectively the concordance between the results of ESBL-E quantitative culture and mPCR/*bla*_CTX-M_ tests, performed on respiratory samples of ESBL-E carriers suspected to have vHAP/VAP. For each micro-organism identification, a result was considered true positive (TP) or true negative (TN) if the results of mPCR and conventional techniques were concordant in that purpose. The conventional cultures were considered as the reference method, i.e., a microorganism identified only by the mPCR and not by the conventional techniques was considered as a false positive (FP), and conversely a target found by the conventional methods and not by the mPCR, was considered a false negative (FN). Agreement between the two methods was assessed by calculating the positive percentage agreement (PPA), and the negative percentage agreement (NPA) rather than sensitivity and specificity as it was difficult to count on standard culture methods as the gold standard [6, 19–21]. PPA was calculated as (TP/(TP + FN)) and NPA as (TN/(TN + FP)). The positive predictive value and the negative predictive value were calculated as 100*(TP/(TP + FP)) and 100*(TN/(TN + FN)), respectively. Accuracy was calculated as (TP + TN)/(TP + TN + FN + FP).

### Impact of mPCR results on initial antibiotic therapy adequacy in ESBL-E carriers with confirmed VAP/vHAP

The secondary objective was to assess retrospectively the impact of using mPCR (mPCR group) on initial antibiotic therapy in ESBL-E carriers with confirmed vHAP/VAP versus conventional diagnostic strategy without mPCR (conventional group). Briefly, mPCR was performed at the physician’s discretion and empirical antibiotic therapy was based on a restrictive antibiotic policy [22] and guidelines [3, 23]. No repetition of mPCR was performed for the same episode. Our ICU protocol for empirical antibiotic therapy is provided in the supplementary methods (Supplementary 1). The clinical impact of mPCR was assessed by the rate of empirical therapies retained as adequate and optimal. Empirical antibiotic therapy referred to the antibiotics prescribed before obtaining quantitative culture results (i.e., after sampling, gram coloration and obtaining mPCR results in the mPCR group, and after sampling and gram coloration in the conventional group). The empirical antibiotic therapy was considered adequate if at least one agent was active against all causative pathogens identified by the conventional microbiological culture, based on AST findings. On the other hand, the therapy was considered optimal if the active agent had the narrowest possible spectrum (Supplementary 1 [24]). The time required to designate optimal antibiotic therapy was defined as the interval between drawing the respiratory sample on which the diagnosis of pneumonia was made, and the initiation of optimal antibiotic therapy, expressed in hours.

### Statistical analysis

Categorical variables, expressed as number (%), were compared using Chi-square or Fisher’s exact tests, whereas continuous variables, expressed as median [25–75th percentile interquartile range (IQR)], were compared using Student’s t-test or Wilcoxon’s rank test, as appropriate. To identify characteristics of episodes associated with adequate empiric antibiotics therapy in patients with confirmed VAP/vHAP, we used multivariable logistic regression. Non-redundant variables selected in bivariate analysis (p < 0.10) and considered clinically relevant were entered into the logistic regression model. To rule out indication biases related to the use of mPCR, multivariable analyses were conducted using overlap propensity-score weighting and propensity-score matching methods. Confounders included in the propensity score were the three following patients’ characteristics recorded at time of sampling: circulatory failure defined as cardiovascular SOFA score of ≥ 3, ratio of partial pressure of arterial oxygen to fraction of inspired oxygen (PaO2/FiO2) of < 150 mmHg, and the use of carbapenem within the 72 h prior to sampling (a known protective factor against ESBL-E pneumonia) [4]. Standardised mean differences were examined to assess balance between groups before and after weighting and matching (eFigure 1). R scripts are provided as supplementary material (Supplementary 1). Statistical significance was defined as P < 0.05. Analyses were computed with IBM SPSS Statistics v22.0 software (IBM Corp, Armonk, NY) and RStudio software, version 4.2.0 (https://www.R-project.org/). The methods and results of this study are presented according to the STROBE guidelines [25].

### Ethical considerations

This observational study was approved by the Institutional Review Board of Henri Mondor university hospital and its database registered by the “*Commission Nationale de l’Informatique et des Libertés*” (n°2,232,944). Patients were informed of their inclusion in the study and written informed consent was waived as per French law.

## Results

**Fig. 1:**
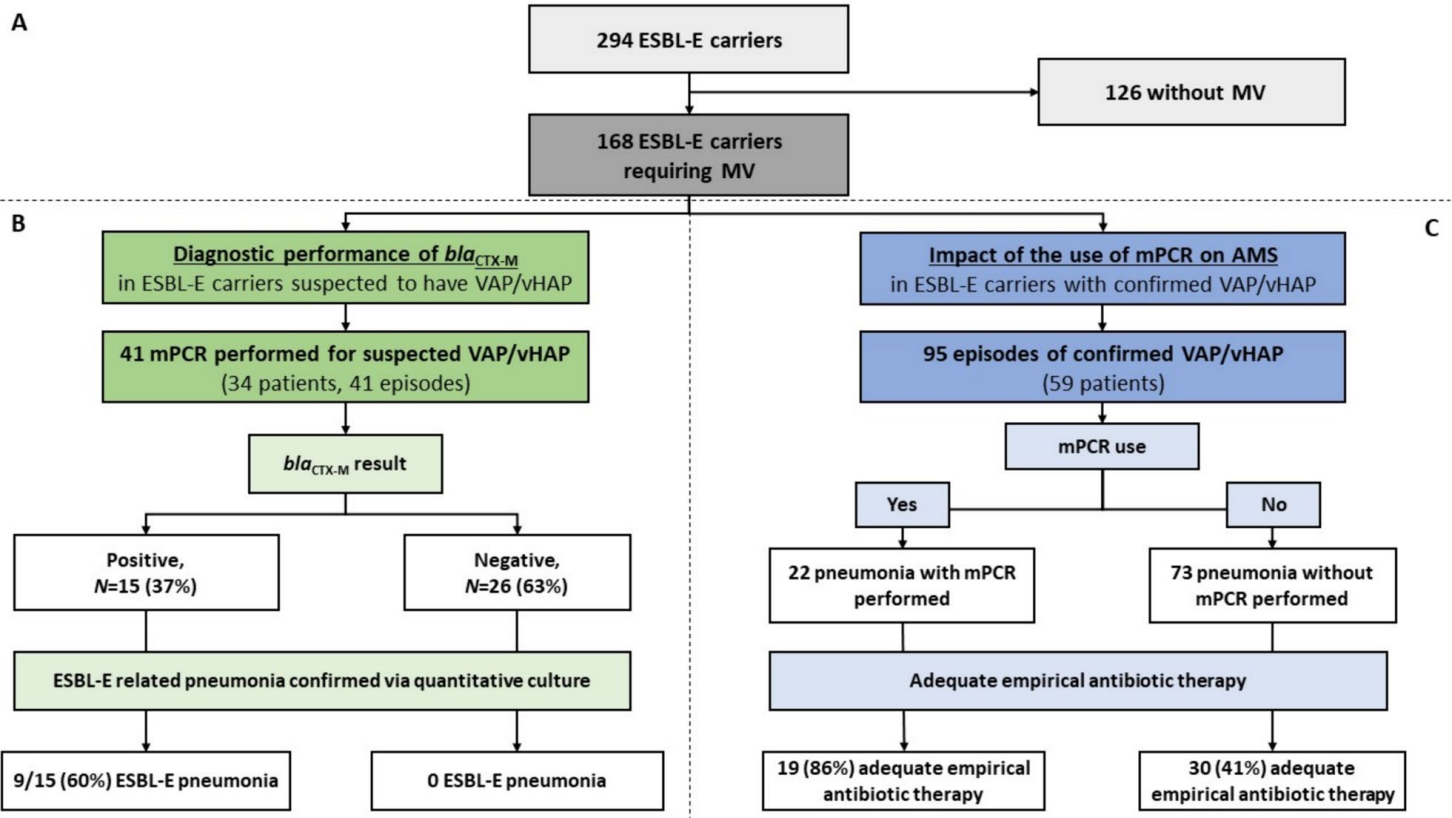
Flow chart of the study. Panel **A** ESBL-E carriers hospitalized in the ICU over the study period. Panel **B** mPCR microbiological performance in ESBL-E carriers with suspected vHAP/VAP. Panel **C** Impact of the use of mPCR on the decision making to initiate antibiotic therapy in ESBL-E carriers with confirmed vHAP/VAP. *CTX-M* Cefotaximase-Munich, *ESBL-E* extended-spectrum β-lactamase-producing *Enterobacterales*, *mPCR* multiplex polymerase chain reaction, *VAP* ventilator associated pneumonia, *vHAP* ventilated hospital-acquired pneumonia

Over the study period, 2827 patients required ICU admission. Of them, 1497 patients had at least one ESBL-E screening by rectal swab, and 294 (10.4%) had a positive rectal swab for ESBL-E. 168 ESBL-E rectal carriers required mechanical ventilation **(**Fig. 1A). The primary endpoint (diagnostic performance of mPCR) was evaluated in 41 suspected episodes of VAP/vHAP (Fig. 1B). The secondary endpoint (impact of performing or not mPCR on initial antibiotic therapy adequacy) was assessed in 95 episodes of quantitative culture-confirmed VAP/vHAP (Fig. 1C).

### Diagnostic performance of mPCR in ESBL-E carriers suspected to have VAP/vHAP

Overall, mPCR was performed on the respiratory samples of 34 of the 168 ESBL-E carriers requiring mechanical ventilation (20%), which represents 41 episodes of suspected VAP/vHAP. The characteristics of the patients (*N* = 34) at ICU admission and those of the episodes (*N* = 41) of suspected pneumonia are respectively reported in eTable 1 and Table 1. mPCR was performed on protected telescopic catheter samples (n = 28/41, 68%), bronchoalveolar lavage fluids (n = 9/41, 22%), and endotracheal aspirates (n = 4/41, 10%). *Bla*_CTX-M_ gene was detected in 15/41 (37%) episodes (Fig. 1). Twenty four episodes (59%) had a positive mPCR, of which 20 (83%) with a definite diagnosis of pneumonia. Among the 17 episodes (41%) with a negative mPCR, 2 (12%) had a definite diagnosis of pneumonia. Assessment of mPCR performance in detecting bacterial and resistance genes in comparison with culture is shown in eTable 2. Overall, the results of quantitative culture and *bla*_CTX-M_ were concordant in 35/41 episodes (85%). Noteworthy, when *bla*_CTX-M_ was negative, culture never found an ESBL-E, suggesting that no pneumonia was due to TEM- or SHV-producing isolates. The six episodes with discordance between genotype (mPCR) and phenotype (culture) are detailed in Supplementary 2. In most episodes (n = 31/41, 76%), the patients were put on empirical antibiotic therapy immediately after drawing the respiratory sample and before having the mPCR results. All of the 24 episodes with positive mPCR were treated with empirical antibiotic therapy after obtaining the mPCR result, and 19 (79%) of them received carbapenems. Of the remaining 17 episodes where mPCR failed to detect bacteria, 11 (65%) received empirical antibiotic therapy, of which 2 (12%) received carbapenems. The latter antibiotics were systematically used whenever the *bla*_CTX-M_ results were positive (n = 15/15, 100%), and spared otherwise in most episodes (n = 20/26, 77%, p < 0.001). An exploratory analysis conducted during the same period on 228 mPCR performed on mechanically ventilated patients with a negative rectal swab for ESBL-E carriage found that mPCR was positive for *bla*_CTX-M_ in two patients (one false positive and one true positive).

**Table 1:** Characteristics of the 41 episodes of suspected vHAP/VAP at the time of BioFire® FilmArray® Pneumonia Panel plus (mPCR)

Variable	All episodes, n = 41
Days after ICU admission	14 [7–21]
Days after mechanical ventilation	11 [4–18]
ESBL-*Enterobacterales* colonisation	
*Escherichia coli* alone	21 (51)
*Klebsiella pneumoniae* and/or *Enterobacter cloacae*	20 (49)
Days after first positive ESBL-E carriage test	6 [3–13]
Previous VAP	17 (41)
Number of previous VAP	1 [1, 2]
Type of suspected episode	
vHAP	6 (15)
VAP	35 (85)
Patient clinical characteristics	
Extracorporeal membrane oxygenation	5 (12)
SOFA score	8 [5–11]
PaO_2_/FiO_2_, mmHg	150 [79–205]
Circulatory failure^1^	26 (63)
Antibiotics received within 72 h prior to mPCR	29 (71)
Carbapenem received within 72 h prior to mPCR	5 (12)

*ESBL-E* extended-spectrum β-lactamase-producing *Enterobacterales*, *ICU* Intensive Care Unit, *mPCR* multiplex polymerase chain reaction, *PaO*_*2*_*/FiO*_*2*_ ratio of the partial pressure of arterial oxygen to the fraction of inspired oxygen, *SOFA* sequential organ failure assessment, *VAP* ventilator associated pneumonia, *vHAP* ventilated hospital-acquired pneumonia

Continuous variables are expressed as median [interquartile range]; categorical variables are expressed as n (%)

^1^Circulatory failure is defined as cardiovascular SOFA score of ≥ 3

### Impact of mPCR use on initial antibiotic therapy adequacy in ESBL-E carriers with confirmed vHAP/VAP

**Fig. 2:**
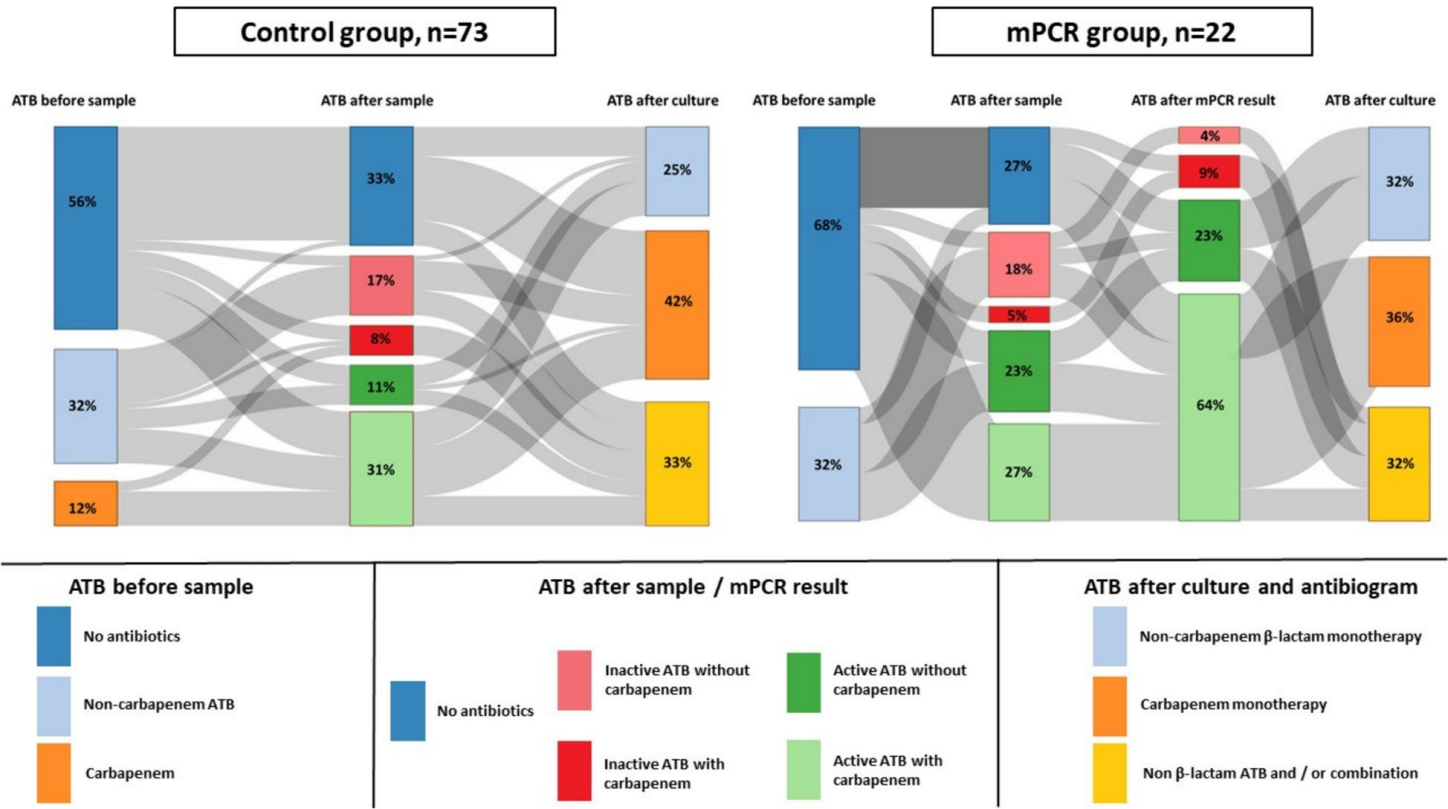
Sankey of diagram of antibiotic stewardship according to the use or not of mPCR in ESBL-E carriers with confirmed vHAP/VAP. *ATB* antibiotic therapy, *ESBL-E* extended-spectrum β-lactamase-producing *Enterobacterales*, *mPCR* multiplex polymerase chain reaction, *MV* mechanical ventilation, *VAP* ventilator associated pneumonia, *vHAP* ventilated hospital-acquired pneumonia

Over the entire study period, 59 ESBL-E carriers developed 95 confirmed vHAP/VAP episodes, of which 22 episodes were tested using mPCR (Fig. 1C). Retrospectively, the identified reasons for not performing mPCR were as follows: the pre-test probability of pneumonia was assessed as low or very low by the clinician in 38 (52%) episodes, a poor quality of sample without leukocytes was present in 11 episodes (15%), 6 episodes (8%) were included at the start of the implementation period of mPCR, and for the remaining 18 episodes (25%), the reason was not reported in the medical record. Patients’ characteristics and organ failure during ICU stay are respectively reported in eTable 3 and Table 2. The mPCR group patients had more circulatory failure, higher SOFA score, and were not put on carbapenem within the 72 h prior to sampling, as compared with their counterparts (Table 2). Forty-seven (49%) vHAP/VAP were related to an ESBL-E, with no difference according to using mPCR [38/73 (52%) vs. 9/22 (41%), p = 0.4] (eTable 4) and 24 (25%) episodes were CRB-induced. The use of empirical antibiotic therapy was not statistically different between mPCR group and conventional group after sampling (Table 3). The empirical antibiotic therapy was more frequently adequate and optimal for vHAP/VAP for patients in the mPCR group, as compared to their counterparts: 19/22 (86%) vs. 30/73 (41%), p < 0.001, and 15/22 (68%) vs. 20/73 (27%), p = 0.001, respectively. This effect was more pronounced in ESBL-E related pneumonia. Sensitivity analyses excluding vHAP, episodes for which carbapenems were administered within the 72 h prior to sampling or including the first episode of pneumonia yielded similar results (Table 3). Figure 2 depicts antibiotic therapy stewardship after sampling and mPCR results. The use of mPCR test, having circulatory failure, and low PaO2/FiO2 ratio were significantly associated with prescription of adequate empirical antibiotic therapy, as shown in the univariate analysis (eTable 5). Alike, mPCR testing was significantly associated with adequate empirical antibiotic therapy in the multivariable logistic regression (adjusted odds ratio (aOR) (95% CI) of 7.5 (2.1–35.9), p = 0.004), propensity-weighting model (aOR of 5.9 (1.6–22.1), p = 0.008), and matching-cohort model (aOR of 5.8 (1.5–22.1), p = 0.01), eTable 6. Results were similar in the sensitivity analysis including only the first pneumonia episode (eTable 7). The time required to shift to optimal antibiotic therapy tended to be shorter for patients in the mPCR group, as compared with their counterparts: 9 [3–45] hours vs. 30 [20–55] hours, p = 0.09 (Table 3, eFigure 2). Similar results were obtained from the sensitivity analysis conducted on only the first pneumonia episode: 24 [3–45] hours vs. 30 [21–50] hours, p = 0.09 (eFigure 2). An exploratory analysis focusing on the first episode of pneumonia (*N* = 59, of which 17 had mPCR testing), found no significant difference in the number of carbapenem treatment days over the seven days following the sampling between mPCR and the conventional groups (2 [0–7] days vs. 2 [0–5] days, *P* = 0.73), even if only ESBL-E non-related cases (*N* = 36, of which 11 had mPCR) were considered (0 [0–2] day vs. 0 [0–1.5] day, *P* = 0.81). Five patients (8.5%) had positive microbiological samples for CRB within the seven days following their first episode of VAP/vHAP: *Stenotrophomonas maltophilia* (protected telescopic catheter *N* = 1, mPCR group; skin culture in a patient with toxic epidermal necrolysis *N* = 1, conventional group), carbapenem-resistant *Pseudomonas aeruginosa (*skin culture in a patient with toxic epidermal necrolysis *N* = 1, conventional group), and *NDM*-producing *Escherichia coli* (protected telescope catheter *N* = 1, mPCR group; urine culture *N* = 1, conventional group).

**Table 2:** Characteristics of the 95 confirmed vHAP/VAP episodes

Variable	Conventional group, n = 73	mPCR group, n = 22	p
Days after admission to the ICU	25 [10–60]	18 [12–38]	0.3
Days after mechanical ventilation	24 [9–59]	13 [9–33]	0.1
ESBL *Enterobacterales* colonisation			
*Escherichia. Coli* alone	32 (44)	11 (50)	0.6
*Klebsiella Pneumoniae* and/or *Enterobacter Cloacae*	36 (49)	11 (50)	1
Others^1^	5 (7)	0	0.6
Days after first positive ESBL-E carriage test	11 [4–29]	9 [3–17]	0.4
Previous VAP	44 (60)	12 (54)	0.6
Number of previous VAP	2 [1–4]	2 [1, 2]	
Antibiotics received within 72 h prior to sampling	45 (62)	12 (54)	0.6
Carbapenem received within 72 h prior to sampling	17 (23)	0	0.01
Type of suspected episode			0.05
vHAP	0	2 (9)	
VAP	73 (100)	20 (91)	
Patient characteristics			
Extracorporeal membrane oxygenation	23 (31)	5 (23)	0.4
SOFA score	6 [4–9]	10 [7–11]	0.007
PaO_2_/FiO_2_, mmHg	151 [83–240]	91 [62–185]	0.1
PaO2/FiO2 < 150 mmHg	35 (48)	14 (64)	0.2
Circulatory failure^2^	30 (41)	15 (68)	0.03
Antibiotic therapy on the day of sampling	32 (44)	7 (32)	0.3
Non-carbapenem β-lactam	23 (31)	7 (32)	1
Carbapenem	9 (12)	0	0.1
Pneumonia characteristics			
ESBL-E related pneumonia	38 (52)	9 (41)	0.4
Carbapenem-resistant pneumonia	19 (26)	5 (23)	0.8

*ESBL-E* extended-spectrum β-lactamase-producing *Enterobacterales*, *ICU* intensive care unit, *mPCR* multiplex polymerase chain reaction, *PaO*_*2*_*/FiO*_*2*_ ratio of the partial pressure of arterial oxygen to the fraction of inspired oxygen, *SOFA* sequential organ failure assessment, *VAP* ventilator associated pneumonia, *vHAP* ventilated hospital-acquired pneumonia

Continuous variables are expressed as median [interquartile range] and compared using Wilcoxon’s rank test; categorical variables are expressed as n (%) and compared using Chi-square or Fisher’s exact tests, as appropriate. No adjustment for multiple comparisons was performed

^1^*Citrobacter Koseri* (n = 1), *Citrobacter Amalonaticus* (n = 1), *Klebsiella Aerogenes* (n = 1), *Klebsiella Oxytoca* (n = 2)

^2^Circulatory failure is defined as cardiovascular SOFA score ≥ 3

**Table 3:** Empirical antibiotic therapy adequation according to the use of mPCR and ESBL-E related pneumonia status in the 95 episodes of nosocomial pneumonia in the mechanically ventilated ESBL-E carriers

All episodes	Conventional group, n = 73	mPCR group, n = 22	p
Empirical antibiotic therapy after sampling			
No initiation	24 (33)	6 (27)	0.6
Non-carbapenem β-lactam	20 (27)	9 (41)	0.2
Carbapenem	29 (40)	7 (32)	0.5
Combination therapy for Gram-negative coverage	20 (27)	10 (45)	0.1
Empirical antibiotic therapy after mPCR result			
No initiation	24 (33)	0	0.002
Non-carbapenem β-lactam	20 (27)	6 (27)	1
Carbapenem	29 (40)	16 (73)	0.007
Combination therapy for Gram-negative coverage	20 (27)	7 (32)	0.7
Antibiotic therapy adequation			
Adequate empirical antibiotic therapy (excluding aminoglycosides)^1^	30 (41)	19 (86)	< 0.001
Adequate empirical antibiotic therapy (including aminoglycosides)^1^	31 (42)	19 (86)	< 0.001
Optimal empirical antibiotic therapy^2^	20 (27)	15 (68)	0.001
Time required for optimal antibiotic therapy, hours	30 [20–55]	9 [3–45]	0.09

ESBL-E related pneumonia	Conventional group, n = 38	mPCR group, n = 9	p
Adequate empirical antibiotic therapy ( excluding aminoglycosides)^1^	13 (34)	9 (100)	< 0.001
Adequate empirical antibiotic therapy ( including aminoglycosides)^1^	14 (37)	9 (100)	< 0.001
Optimal empiric antibiotic therapy^2^	13 (34)	9 (100)	0.001

Non ESBL-E related pneumonia	Conventional group, n = 35	mPCR group, n = 13	p
Adequate empirical antibiotic therapy (excluding aminoglycosides)^1^	17 (49)	10 (77)	0.08
Adequate empirical antibiotic therapy (including aminoglycosides)^1^	17 (49)	10 (77)	0.08
Optimal empirical antibiotic therapy^2^	7 (20)	6 (46)	0.1
Overconsumption of carbapenem^3^	7 (20)	7 (54)	0.03

Ventilator-associated pneumonia	Conventional group, n = 73	mPCR group, n = 20	p
Adequate empirical antibiotic therapy (including aminoglycosides)^1^	31 (42)	18 (90)	< 0.001

Patients without carbapenem within 72 h prior to sample	Conventional group, n = 56	mPCR group, n = 22	p
Adequate empirical antibiotic therapy (including aminoglycosides)^1^	21 (37)	19 (86)	< 0.001

First episode of VAP/vHAP	Conventional group, n = 42	mPCR group, n = 17	p
Adequate empirical antibiotic therapy (including aminoglycosides)^1^	17 (40)	15 (88)	< 0.001

*ESBL-E* extended-spectrum β-lactamase-producing *Enterobacterales*, *mPCR* multiplex polymerase chain reaction

Categorical variables are expressed as n (%) and compared using Chi-square or Fisher’s exact tests as appropriate. No adjustment for multiple comparisons was performed

^1^Empirical antibiotic therapy was considered adequate if at least one agent was active on all of the offensive pathogens identified by the conventional microbiological culture, based on antibiotic susceptibility findings

^2^Empirical antibiotic therapy was considered optimal if it was not only active but also not excessively broad-spectrum

^3^Overconsumption of carbapenem was defined as an empirical use of carbapenem whenever the causative bacteria was susceptible to a first-line β-lactam

## Discussion

To the best of our knowledge, we herein report the first study on mPCR testing specifically focused on ESBL-E carriers, with the following main results: (i) in suspected vHAP/VAP, *bla*_CTX-M_ had an excellent concordance with standard culture to rule out ESBL-E-related pneumonia; (ii) in confirmed vHAP/VAP, an mPCR-based approach significantly increased the rate of prescribing adequate and optimal empirical antibiotic therapy in the specific context of our ICU with a restrictive antibiotic policy. AMS for suspected vHAP/VAP in ESBL-E carriers is a daily challenge for intensivists who need to choose the most likely active antibiotic to give in case pneumonia settles [26], and to decide which episodes to treat, since ventilator-associated events mostly reflect non-infectious events [4].

The overall diagnostic value of mPCR we observed is consistent with previous studies findings [6–10]. The reported concordance of negative *bla*_CTX-M_ result with the culture helps to eliminate ESBL-E-induced pneumonia and consequently to serenely spare carbapenems upon dealing with suspected vHAP/VAP in ESBL-E carriers. Multicenter studies using Biofire® Filmarray® also reported a 100% negative concordance of *bla*_CTX-M_ to rule out the diagnosis of ESBL-E related pneumonia, but included very few of such cases [7, 8]. mPCR approach is entangled by with two inherent limitations: (i) the risk of false negatives generated by *Enterobacterales* that are not included in the mPCR panel [6]; (ii) its inadequacy in countries where *bla*_*CTX-M*_ is not the predominant gene expressed by ESBL-E.

AMS is a challenging but crucial matter in ICU, especially in ESBL-E carriers. Generalising prescription of carbapenems to ESBL-E carriers is not a suitable approach for several reasons. First, as previously observed, a quarter of pneumonia cases CRB-induced [27]. Second, unnecessary exposure to carbapenems multiplies the risk of triggering CRB in future infections [28–30]. Third, recent studies described a positive impact of a restrictive antibiotic policy [22, 31]. In our study, confirmed VAP accounted for less than half, and ESBL-E-related VAP for less than a quarter of the suspected pneumonia episodes, which is in line with previous reports [4]. mPCR use could therefore guide decision-making process for AMS in ESBL-E carriers, especially when physician decided to initiate antibiotic therapy for whom guidelines recommend the use of carbapenems as empirical antibiotic therapy [3], (i) by enhancing a reasonable restrictive AMS policy that precludes carbapenems facing suspected VAP/vHAP, thanks to the high reported performance value of *bla*_CTX-M_ to rule out the diagnosis of ESBL-E related pneumonia; (ii) by increasing the rate of prescribing adequate and optimal empirical antibiotic therapy in confirmed VAP/vHAP. However, if mPCR is used in very low pre-test clinical probability of pneumonia, due to the high sensitivity and the rate of overdiagnosed pneumonia, the risk of overconsumption of carbapenem may prevail. An algorithm for the use of mPCR in ESBL-E carriers with a suspected VAP/vHAP is proposed in eFigure 3. Nonetheless and given the conflicting results recently reported by randomised controlled trials on mPCR [11, 32], the impact mostly pronounced in the initial hours following respiratory sampling and the cost of individual tests, the role of mPCR in AMS for ICU patients needs further investigations. Indeed, most studies using mPCR showed no difference in number of days alive and free from antibiotics or the duration of use of broad spectrum antibiotics [11, 12]. A promising area of application could be specific situations, such as patients at risk from MDR bacteria.

Our study has several limitations. First, it is monocentric with a small number of patients, which implies a cautious interpretation of our findings. These results need to be confirmed by large multicentre studies including ICUs with various local ecology and antibiotic policy. Our findings are not applicable in regions with ESBL-E mainly due to TEM- or SHV-producing isolates. Second, the inclusion of multiple episodes related to the same patient might be a source of bias, but results were similar in the sensitivity analysis including only the first pneumonia episode. Third, mPCR was performed at the physician’s discretion resulting in an imbalance in some important variables (shock, exposure to carbapenems) between the mPCR and conventional groups. However, we present a real-life picture of an mPCR-based AMS focused on this high-risk ICU population. In addition, the propensity-weighting, the matching-cohort, and the multivariable logistic regression models showed that the mPCR-based approach was independently associated with better antibiotic stewarding towards more adequate and optimal empirical antibiotic therapy. Yet, the use of these models in a small sample needs to be interpreted cautiously. Fourth, in our study, we did not provide data on the cost effectiveness and the ecological impacts of such an approach. These results are preliminary and need to be evaluated in prospective randomised clinical trials. The latter will have to evaluate the ecological impact of a mPCR-based AMS (i.e., antibiotic resistance rates, carbapenems consumption) and the cost-effectiveness of such an approach.

## Conclusion

mPCR *bla*_CTX-M_ showed an excellent diagnostic value to rule out the diagnosis of ESBL-E related pneumonia in ESBL-E carriers with suspected VAP/vHAP. The secondary analysis of the use of mPCR in confirmed VAP/vHAP found that a mPCR-based approach was associated with increased prescription of adequate empirical antibiotic therapy. Performing mPCR on respiratory samples seems to be a promising tool in ESBL-E carriers with suspected vHAP/VAP. However, if mPCR is used in very low pre-test clinical probability of pneumonia, due to the high sensitivity and the rate of overdiagnosed pneumonia, the risk of overconsumption of carbapenem may prevail. Further studies are warranted.

### Supplementary Information

Supplementary Material 1.

Supplementary Material 2.

Supplementary Material 3. eFigure 1. Propensity score balance. Comparisons of the absolute standardised mean differences on selected covariates (circulatory failure defined as cardiovascular SOFA score ≥ 3, PaO2/FiO2 < 150 mmHg and the use of carbapenem within 72 h prior to sample), before and after weighting and matching.

Supplementary Material 4. eFigure 2. Kaplan-Meier curve of the proportion of patients receiving optimal antibiotic therapy according to whether mPCR was used or not (censoring threshold: 48 h). A. Kaplan-Meier Curve for optimal antibiotic therapy according to the use of mPCR in the whole cohort (*N* = 95). P-value was determined using the log-rank test. B. Kaplan-Meier Curve for optimal antibiotic therapy according to the use of mPCR in the first episode of pneumonia (*N* = 59). P-value was determined using the log-rank test.

Supplementary Material 5. eFigure 3. Proposed Algorithm for empiric antibiotic therapy in ESBL-E carriers with a suspicion of VAP. CTX-M, Cefotaximase-Munich; ESBL-E, extended-spectrum β-lactamase-producing *Enterobacterales*; mPCR, multiplex polymerase chain reaction; VAP, ventilator associated pneumonia.

Supplementary Material 6.

## Data Availability

The datasets used and/or analysed during the current study are available from the corresponding author on reasonable request.

## Abbreviations

AMS: Antimicrobial stewardship
ARDS: Acute respiratory distress syndrome
AST: Antibiotic susceptibility testing
CRB: Carbapenem-resistant bacteria
ESBL-E: Extended-spectrum β-lactamase-producing *Enterobacterales*
FN: False negative
FP: False positive
ICU: Intensive care unit
MDR: Multidrug-resistant
mPCR: Multiplex PCR
MV: Mechanical ventilation
SAPS: Simplified acute physiology score
SOFA: Sequential organ failure assessment
TN: True negative
TP: True positive
VAP: Ventilator-associated pneumonia
vHAP: Ventilated hospital-acquired pneumonia
CTX-M: Cefotaximase-Munich
NPA: Negative percentage agreement
PPA: Positive percentage agreement
