# Supplementary material for: Performance and impact of rapid multiplex PCR on diagnosis and treatment of ventilated hospital-acquired pneumonia in patients with extended-spectrum β-lactamase-producing Enterobacterales rectal carriage
Source: Ann Intensive Care. 2024 Jul 29;14:118. doi: 10.1186/s13613-024-01348-5 (PMC11286905; doi:10.1186/s13613-024-01348-5)
Supplement: Supplementary file 1 — Supplementary Material 1. [file 13613_2024_1348_MOESM1_ESM.docx]

**SUPPLEMENTARY 1**

# Empirical antibiotic therapy for suspected VAP/vHAP

In the absence of signs of severity (shock, acute respiratory distress syndrome (ARDS), or being immunocompromised), it is possible to wait for direct examination and culture results before prescribing antibiotics.

## Suspected VAP/vHAP in patients hospitalized for less than 5 days, without prior antibiotic therapy, ARDS or *Pseudomonas aeruginosa* colonisation

##

- Amoxicillin-clavulanic acid or third-generation cephalosporin (cefotaxime), inactive against *P. aeruginosa*

## Suspected VAP/vHAP in patients hospitalized for more than 5 days or with prior antibiotic therapy

- Patients with negative ESBL-E rectal colonisation :
  - First episode of VAP: Cefepime; if septic shock or immunocompromised or ARDS add Amiklin, (piperacillin-tazobactam is rather used as a second line)
  - If there was a previous VAP, the bacteria identified then and any resistance must be taken into account.
- ESBL-E carriers
  - Request mPCR whenever possible.
  - If mPCR is not available, meropenem.

# Optimal empirical antibiotic therapy

The antibiotic therapy was considered optimal if its active agent had the narrowest possible spectrum, and whose classification was based on a ranking of β-lactams according to both their spectra and their global ecological consequences (six-rank classification), published by Weiss et al. (1). This classification had already been used in a previous article investigating the impact of mPCR (2).


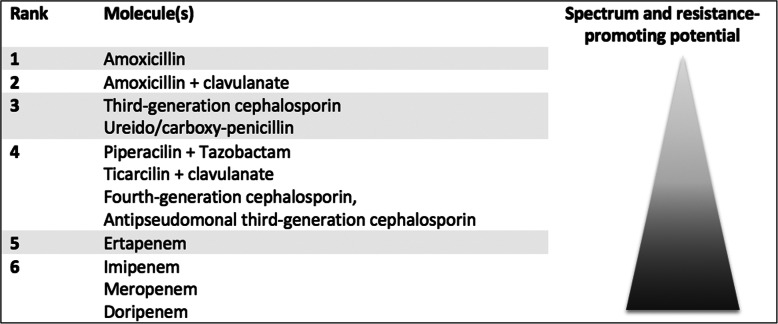


Consensual ranking of *β*-lactams according to both their spectrum and their resistance-promoting potential according to Weiss et al., *Clinical Microbiology and Infection* 2015 “Elaboration of a Consensual Definition of De-Escalation Allowing a Ranking of β-Lactams”

# R scripts: propensity scores

## Package used for propensity score

library(PSweight)

## Setting the covariables

ps.formula <- mPCR ~ Circulatory_Failure + PF_inf150 + Carba_72h

## SMD Graph

# Determine methods

spstat <- SumStat(ps.formula = ps.formula, data = d,

weight=c("overlap","matching"))

# Plot density graph

plot(spstat, type="density")

# Plot SMD Graph

rownames(spstat$unweighted.sumstat) = c("Circu. Fail.","PF <150","Carba 72h")

plot(spstat, metric="ASD")

spstat

## Overlap Weighting

# Setting the model

sptest.ow <- PSweight(ps.formula = ps.formula, family = 'binomial',

yname = 'atb_opt_on', data = d, weight = 'overlap')

# ATO

summary(sptest.ow)

# P0 mean per group

print(sptest.ow$muhat)

# OR

contrasts.mult <- rbind(c(-1,1))

sum.sptest.ow.or <- summary(sptest.ow, type = "OR", contrast = contrasts.mult)

exp(sum.sptest.ow.or$estimates[c(1,4,5)])

# pvalue OR

sum.sptest.ow.or

## Matching

# Setting the model

sptest.m <- PSweight(ps.formula = ps.formula, family = 'binomial',

yname = 'atb_opt_on', data = d, weight = 'matching')

# ATM

summary(sptest.m)

# P0 mean per group

print(sptest.m$muhat)

# OR

contrasts.mult <- rbind(c(-1,1))

sum.sptest.m.or <- summary(sptest.m, type = "OR", contrast = contrasts.mult)

exp(sum.sptest.m.or$estimates[c(1,4,5)])

# pvalue OR

sum.sptest.m.or

# References

1. Weiss E, Zahar JR, Lesprit P, Ruppe E, Leone M, Chastre J, et al. Elaboration of a consensual definition of de-escalation allowing a ranking of β-lactams. Clin Microbiol Infect Off Publ Eur Soc Clin Microbiol Infect Dis. 2015 Jul;21(7):649.e1-10.

2. Monard C, Pehlivan J, Auger G, Alviset S, Tran Dinh A, Duquaire P, et al. Multicenter evaluation of a syndromic rapid multiplex PCR test for early adaptation of antimicrobial therapy in adult patients with pneumonia. Crit Care. 2020 Jul 14;24(1):434.
