# Supplementary material for: Performance and impact of rapid multiplex PCR on diagnosis and treatment of ventilated hospital-acquired pneumonia in patients with extended-spectrum β-lactamase-producing Enterobacterales rectal carriage
Source: Ann Intensive Care. 2024 Jul 29;14:118. doi: 10.1186/s13613-024-01348-5 (PMC11286905; doi:10.1186/s13613-024-01348-5)
Supplement: Supplementary file 2 — Supplementary Material 2. [file 13613_2024_1348_MOESM2_ESM.docx]

**SUPPLEMENTARY 2**

**Characteristics and outcomes of the suspected VAP/vHAP cases with positive bla_CTX-M_ result (*N*=6/15, 40%) and negative ESBL-E related pneumonia as confirmed by culture.**

| **Case** | **Previous ESBL-related pneumonia before this episode** | **Sampling technique** | **Antibiotic therapy prescribed at the time of sampling** | **mPCR result** | **Conventional culture result** | **Carbapenem after**  **mPCR resuls** | Definitive Antibiotic therapy | Carbapenem overconsumption | Outcomes | ESBL-E related VAP after this episode |
| --- | --- | --- | --- | --- | --- | --- | --- | --- | --- | --- |
| 1 | No | PTC | None | 10.5 *E. cloacae*  10.7 *E. coli*  10.4 *K. pneumonia*  *bla*_CTX-M_ + | 10.6 *C. striatum*  10.6 *E. coli* | Yes | Despite the presence of wild-type *E. coli*, the patient received Meropenem due to a ECMO cannula infection related to *E. cloacae* ESBL. | No  Extra respiratory infection | Early recurrence of ESBL-E (*K. Pneumoniae*) VAP 48 hours after Meropenem discontinuation | Yes; ESBL-related VAP at day 25, two days after discontinuation of Meropenem |
| 2 | No | BAL | Imipenem (initiation 24 hours before sampling) | 10.6 *E. coli*  *bla*_CTX-M_ + | Negative | Yes | The patient received Imipenem as treatment. | No  Probable ESBL-related pneumonia as sample was performed 24h after antibiotic initiation | Noteworthy, it was a vHAP episode, and all the respiratory samples were collected after the patient had already received antibiotic therapy. The patient's condition improved with the treatment. | No |
| 3 | No | BAL | Piperacillin-Tazobactam | 10.5 *K. pneumonia*  *bla*_CTX-M_ + | Negative | Yes | Meropenem was discontinued upon receiving the conventional culture results. | Yes | The patient's condition improved without new episode of VAP | No |
| 4 | No | PTC | Cefepime | 10.7 *P. aeruginosa*  10.4 *S. pneumonia*  *bla*_CTX-M_ + | 10.6 *P. aeruginosa* | Yes | Antibiotic therapy was de-escalated to piperacillin-ciprofloxacin upon receiving the conventional culture and antibiotic susceptibility test results. | Yes | 7 days after, the patient died of refractory COVID-19 related ARDS. No ESBL-E related infection was identified on follow-up respiratory samplings. | No |
| 5 | No | ETA | None | 10.7 *S. agalactiae*  10.7 *E. cloacae*  bla_NDM_  *bla*_CTX-M_ + | 10.6 *S. agalactiae*  10.7 *R. ornithinolytica*  < 10.2 *E. coli (*ESBL plus NDM) | yes | Empirical antibiotic therapy was de-escalated to amoxicillin plus Clavulanate upon receiving the results of conventional culture and antibiotic susceptibility test. | Yes | The evolution of this episode of VAP was favourable. | The patient subsequently developed ESBL E-related VAP at day 36 (3 weeks after the completion of treatment with amoxicillin plus Clavulanate). |
| 6 | Yes  E. coli ESBL-related VAP 15 days before | ETA | Piperacillin-Tazobactam | 10.7 *P. aeruginosa*  *bla*_CTX-M_ + | 10.7 *P. aeruginosa* | Yes | The patient received meropenem plus amikacin as empirical antibiotic therapy. | Yes | The outcome was unfavourable, resulting in death within 48 hours due to multi-organ failure | No |
| Abbreviations: BAL, bronchoalveolar lavage; ESBL-E, extended-spectrum β-lactamase-producing *Enterobacterales*; ETA, endotracheal aspirate; PTC, protected telescope catheter | | | | | | | | | | |
