# Supplementary figures and images for: Performance and impact of rapid multiplex PCR on diagnosis and treatment of ventilated hospital-acquired pneumonia in patients with extended-spectrum β-lactamase-producing Enterobacterales rectal carriage
Source: Ann Intensive Care. 2024 Jul 29;14:118. doi: 10.1186/s13613-024-01348-5 (PMC11286905; doi:10.1186/s13613-024-01348-5)

## Slide 1
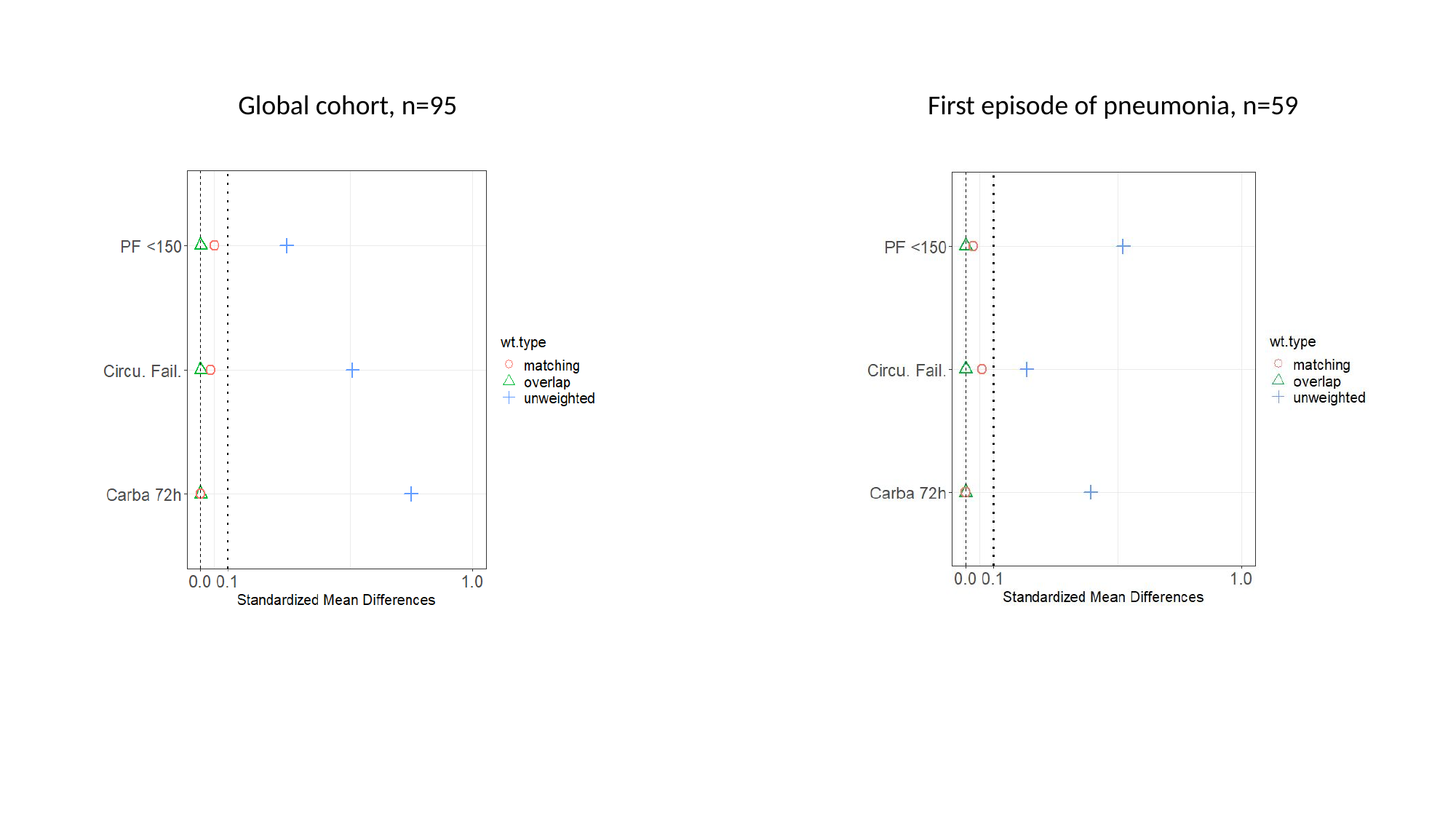

Global cohort, n=95
First episode of pneumonia, n=59

Supplement: Supplementary file 3 — Supplementary Material 3. eFigure 1. Propensity score balance. Comparisons of the absolute standardised mean differences on selected covariates (circulatory failure defined as cardiovascular SOFA score ≥ 3, PaO2/FiO2 < 150 mmHg and the use of carbapenem within 72 h prior to sample), before and after weighting and matching. [file 13613_2024_1348_MOESM3_ESM.pptx]
