# Supplementary material for: Performance and impact of rapid multiplex PCR on diagnosis and treatment of ventilated hospital-acquired pneumonia in patients with extended-spectrum β-lactamase-producing Enterobacterales rectal carriage
Source: Ann Intensive Care. 2024 Jul 29;14:118. doi: 10.1186/s13613-024-01348-5 (PMC11286905; doi:10.1186/s13613-024-01348-5)
Supplement: Supplementary file 4 — Supplementary Material 4. eFigure 2. Kaplan-Meier curve of the proportion of patients receiving optimal antibiotic therapy according to whether mPCR was used or not (censoring threshold: 48 h). A. Kaplan-Meier Curve for optimal antibiotic therapy according to the use of mPCR in the whole cohort (N = 95). P-value was determined using the log-rank test. B. Kaplan-Meier Curve for optimal antibiotic therapy according to the use of mPCR in the first episode of pneumonia (N = 59). P-value was determined using the log-rank test. [file 13613_2024_1348_MOESM4_ESM.pptx]

## Slide 1
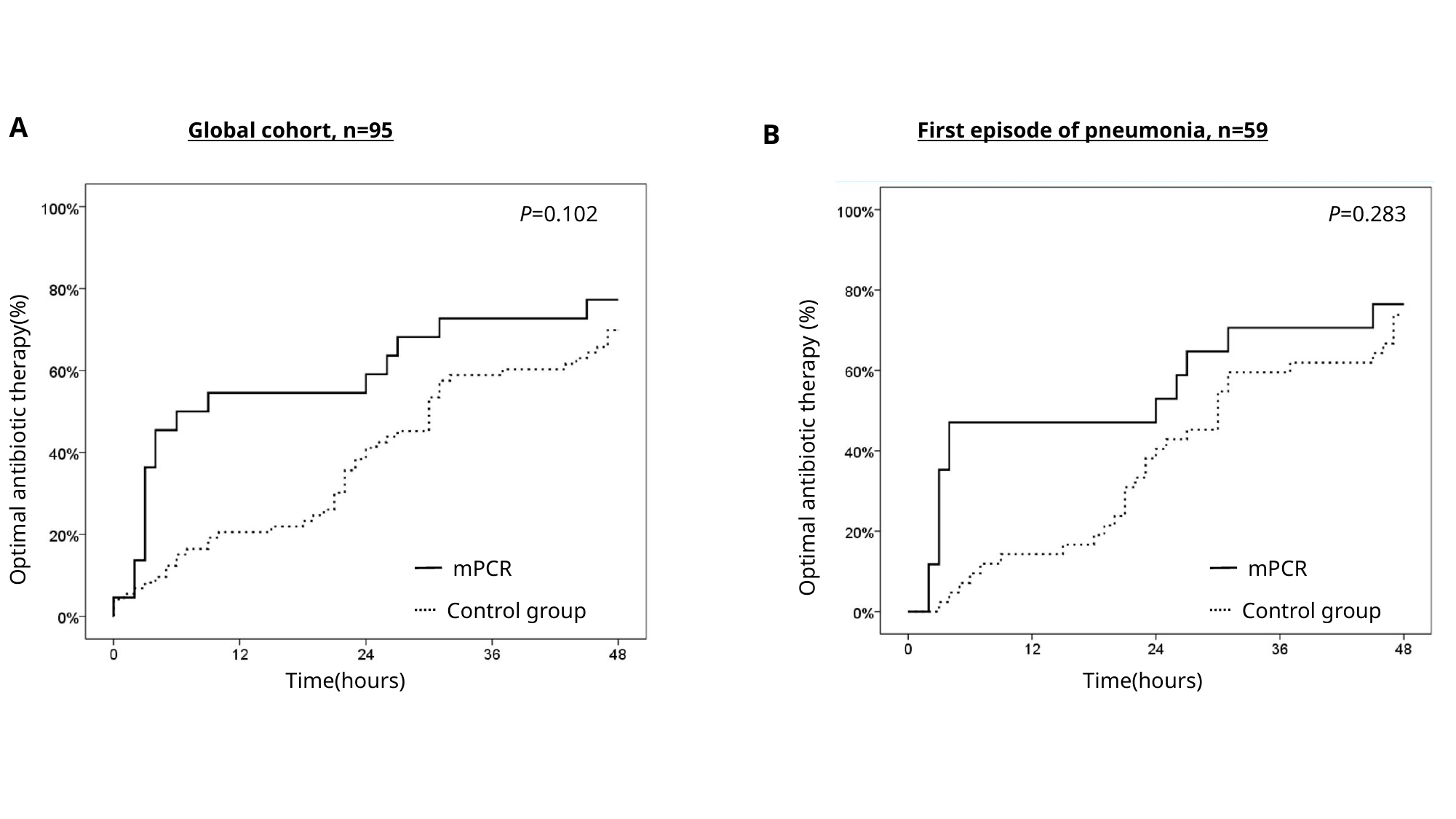

A
Global cohort, n=95
B
First episode of pneumonia, n=59
P=0.102
P=0.283
Optimal antibiotic therapy(%)
Optimal antibiotic therapy (%)
mPCR
Control group
mPCR
Control group
Time(hours)
Time(hours)
