# Supplementary material for: Performance and impact of rapid multiplex PCR on diagnosis and treatment of ventilated hospital-acquired pneumonia in patients with extended-spectrum β-lactamase-producing Enterobacterales rectal carriage
Source: Ann Intensive Care. 2024 Jul 29;14:118. doi: 10.1186/s13613-024-01348-5 (PMC11286905; doi:10.1186/s13613-024-01348-5)
Supplement: Supplementary file 5 — Supplementary Material 5. eFigure 3. Proposed Algorithm for empiric antibiotic therapy in ESBL-E carriers with a suspicion of VAP. CTX-M, Cefotaximase-Munich; ESBL-E, extended-spectrum β-lactamase-producing Enterobacterales; mPCR, multiplex polymerase chain reaction; VAP, ventilator associated pneumonia. [file 13613_2024_1348_MOESM5_ESM.pptx]

## Slide 1
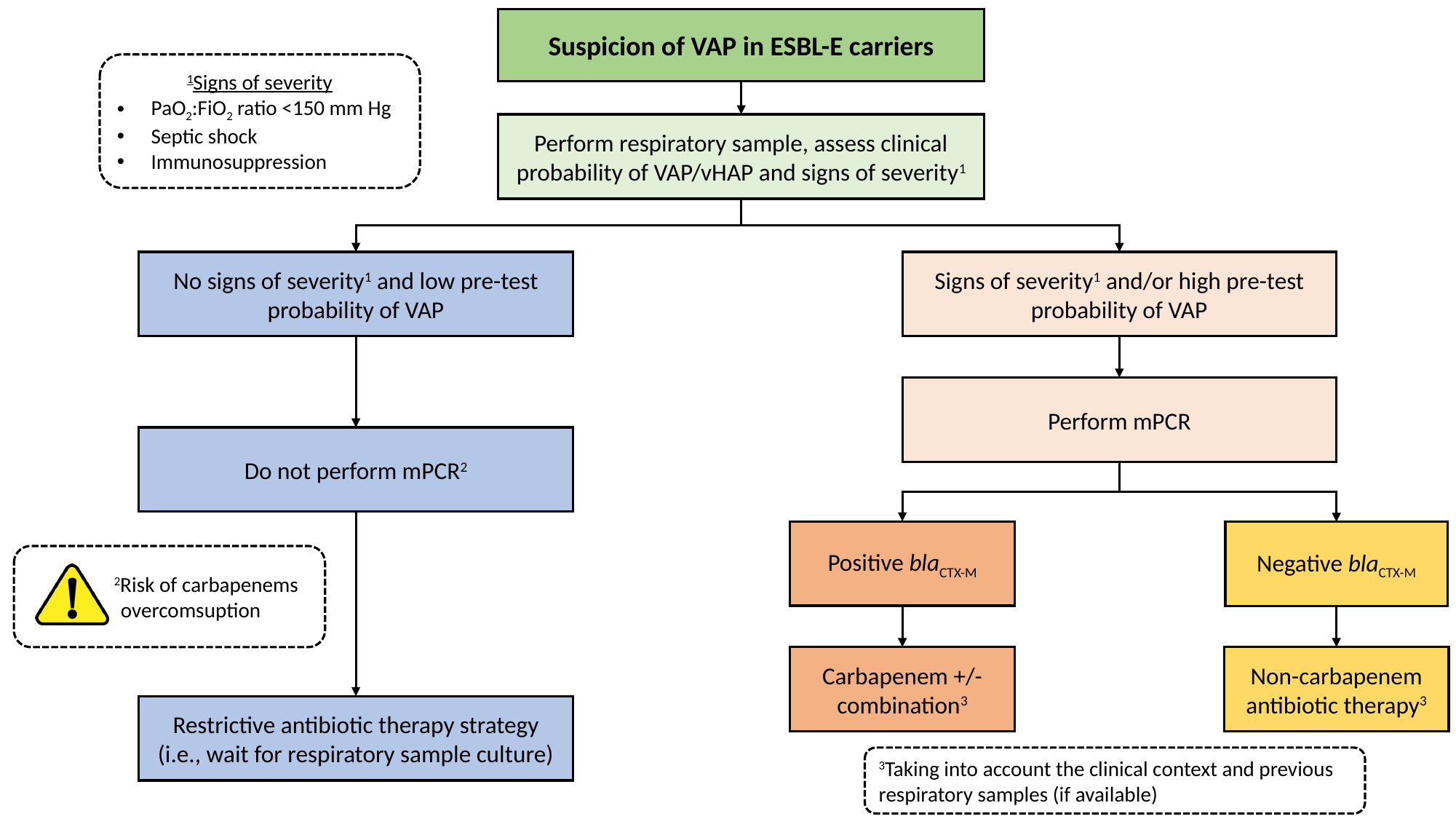

Suspicion of VAP in ESBL-E carriers
1Signs of severity
PaO2:FiO2 ratio <150 mm Hg
Septic shock
Immunosuppression
Perform respiratory sample, assess clinical probability of VAP/vHAP and signs of severity1
No signs of severity1 and low pre-test probability of VAP
Signs of severity1 and/or high pre-test probability of VAP
Perform mPCR
Do not perform mPCR2
Positive blaCTX-M
Negative blaCTX-M
 2Risk of carbapenems
 overcomsuption
Carbapenem +/- combination3
Non-carbapenem antibiotic therapy3
Restrictive antibiotic therapy strategy (i.e., wait for respiratory sample culture)
3Taking into account the clinical context and previous respiratory samples (if available)
