# Supplementary material for: Performance and impact of rapid multiplex PCR on diagnosis and treatment of ventilated hospital-acquired pneumonia in patients with extended-spectrum β-lactamase-producing Enterobacterales rectal carriage
Source: Ann Intensive Care. 2024 Jul 29;14:118. doi: 10.1186/s13613-024-01348-5 (PMC11286905; doi:10.1186/s13613-024-01348-5)
Supplement: Supplementary file 6 — Supplementary Material 6. [file 13613_2024_1348_MOESM6_ESM.docx]

| **eTable 1. Characteristics of the 34 patients with suspected vHAP/VAP at ICU admission** | |
| --- | --- |
| **Variable** |  |
| Age, years | 58 [48-66] |
| Women | 7 (21) |
| Body mass index, kg/m² | 24 [23-33] |
| SAPS II at ICU admission | 40 [29-61] |
| **Comorbidities** |  |
| Charlson Comorbidity index | 1 [0-3] |
| Hypertension | 17 (50) |
| Diabetes mellitus | 14 (41) |
| Congestive heart failure (NYHA 3–4) | 2 (6) |
| COPD | 1 (3) |
| Immunosuppressive condition^1^ | 12 (35) |
| COVID-19 related admission | 21 (62) |
| **Organ failure & management during ICU stay** |  |
| Vasopressors | 31 (91) |
| Renal replacement therapy | 17 (50) |
| Acute respiratory distress syndrome | 27 (80) |
| Extracorporeal membrane oxygenation | 8 (23) |
| **Outcomes** |  |
| ICU length of stay, days | 38 [20-63] |
| In-ICU mortality | 15 (44) |
| Abbreviations: ESBL-E, extended-spectrum β-lactamase-producing *Enterobacterales*; ICU, Intensive Care Unit; SASP, simplified acute physiology score; VAP, ventilator associated pneumonia; vHAP, ventilated hospital-acquired pneumonia  Continuous variables are expressed as median [interquartile range]; categorical variables are expressed as n (%).  ^1^Cancer (n=1), blood malignancy (n=7), solid organ transplant (n=6), HIV infection (n=1), immunosuppressant (n=1) | |

| **eTable 2. Analysing performance of BioFire® FilmArray® Pneumonia plus Panel compared to culture, taking into account microbiological thresholds** | | | | | | | | | | |
| --- | --- | --- | --- | --- | --- | --- | --- | --- | --- | --- |
|  | **Organisms** | **culture+/mPCR+** | **mPCR +/culture −** | **Culture +/mPCR −** | **Culture −/mPCR −** | **PPA^1^ (%)** | **NPA^1^ (%)** | **PPV (%)** | **NPV (%)** | **Accuracy** |
| **GRAM +** | *Streptococcus pneumoniae* | 0 | 1 | 0 | 40 | n.a | 98 | 0 | 100 | n.a |
|  | *Staphylococcus aureus* | 5 | 1 | 0 | 35 | 100 | 97 | 83 | 100 | 98 |
|  | *Streptococcus pyogenes* | 0 | 0 | 0 | 41 | n.a | 100 | n.a | 100 | n.a |
|  | *Streptococcus agalactiae* | 1 | 1 | 0 | 39 | 100 | 98 | 50 | 100 | 98 |
|  | **TOTAL** | **6** | **3** | **0** | **155** | **100**  **[54-100]** | **98**  **[95-100]** | **67**  **[30-93]** | **100**  **[98-100]** | **98**  **[95-100]** |
| **GRAM –** | *Acineterobacter calcoaceticus-baumannii* complex | 1 | 1 | 0 | 39 | 100 | 98 | 50 | 100 | 98 |
|  | *Enterobacter cloacae* complex | 5 | 2 | 2 | 32 | 71 | 94 | 71 | 94 | 90 |
|  | *Escherichia coli* | 3 | 3 | 0 | 35 | 100 | 92 | 50 | 100 | 93 |
|  | *Haemophilus influenzae* | 1 | 0 | 0 | 40 | 100 | 100 | 100 | 100 | 100 |
|  | *Klebsiella aerogenes* | 0 | 0 | 0 | 41 | n.a | 100 | n.a | 100 | n.a |
|  | *Klebsiella oxytoca* | 0 | 0 | 0 | 41 | n.a | 100 | n.a | 100 | n.a |
|  | *Klebsiella pneumoniae* group | 3 | 2 | 1 | 35 | 75 | 95 | 60 | 97 | 93 |
|  | *Moraxella catarrhalis* | 0 | 1 | 0 | 40 | n.a | 98 | 0 | 100 | n.a |
|  | *Proteus*spp. | 0 | 1 | 0 | 40 | n.a | 98 | 0 | 100 | n.a |
|  | *Pseudomonas aeruginosa* | 8 | 0 | 1 | 32 | 89 | 100 | 100 | 97 | 98 |
|  | *Serratia marcescens* | 0 | 0 | 0 | 41 | n.a | 100 | n.a | 100 | n.a |
|  | **TOTAL** | **21** | **10** | **4** | **416** | **84**  **[64-95]** | **98**  **[96-99]** | **68**  **[49-83]** | **99**  **[98-100]** | **97**  **[95-98]** |
| **TOTAL** | | **27** | **13** | **4** | **571** | **87**  **[70-96]** | **98**  **[96-99]** | **67**  **[51-81]** | **99**  **[98-100]** | **97**  **[96-98]** |
| **VIRUS^2^** | |  |  |  |  |  |  |  |  |  |
| **RESISTANCE** | |  |  |  |  |  |  |  |  |  |
| **CTX-M^3^** | | **9** | **6** | **0** | **26** | **100**  **[66-100]** | **81**  **[64-93]** | **60**  **[32-84]** | **100**  **[87-100]** | **85**  **[71-94]** |
| MecA | | 1 | 0 | 0 | 40 | 100 | 100 | 100 | 100 | 100 |
| OXA-48^4^ | | 0 | 0 | 0 | 41 | n.a | 100 | n.a | 100 | n.a |
| VIM^4^ | | 0 | 0 | 0 | 41 | n.a | 100 | n.a | 100 | n.a |
| KPC^4^ | | 0 | 0 | 0 | 41 | n.a | 100 | n.a | 100 | n.a |
| NDM^4^ | | 0 | 1^5^ | 0 | 40 | n.a | 98 | 0 | 100 | n.a |
| IMP^4^ | | 0 | 0 | 0 | 41 | n.a | 100 | n.a | 100 | n.a |
| **TOTAL** | | **10** | **7** | **0** | **270** | **100**  **[69-100]** | **97**  **[94-99]** | **56**  **[31-78]** | **100**  **[99-100]** | **98**  **[95-99]** |
| Abbreviations: mPCR, multiplex polymerase chain reaction; NPA, negative percentage agreement, PPA, positive percentage agreement  ^1^In the case of LRTIs, it is difficult to depend on standard culture methods as gold standard, thus the terms PPA and NPA are more commonly used.  ^2^BioFire® FilmArray® Pneumonia plus Panel did not identify any virus among its targets.  ^3^ESBL were phenotypically detected on antimicrobial susceptibility testingif a difference of more than 5 mm between the discs “cefepime” and “cefepime + clavulanate” was observed and/or using a double-disk synergy test and was considered as gold standard  ^4^Carbapenemases were confirmed by qualitative lateral flow immunoassay (NG-Test® CARBA-5, NG-Biotech, Guipry, France) and was considered as gold standard  ^5^One patient with < 10.2 E. coli (ESBL plus NDM) in endotracheal aspirate culture considered discordant because was below microbiological thresholds | | | | | | | | | | |

| **eTable 3. Characteristics of the mechanically ventilated ESBL-E carriers with confirmed ventilator associated pneumonia or ventilated hospital-acquired pneumonia according to the use of mPCR** | | |
| --- | --- | --- |
| **Variable** | **Conventional group, n=45^1^** | **mPCR group, n=20^1^** |
| Age, years | 59 [49-66] | 57 [48-67] |
| Women | 14 (31) | 6 (30) |
| Body mass index, kg/m² | 29 [25-34] | 29 [24-34] |
| SAPS II at ICU admission | 43 [31-63] | 44 [30-65] |
| **Comorbidities** |  |  |
| Charlson Comorbidity index | 1 [0-1] | 1 [0-3] |
| Hypertension | 25 (56) | 10 (50) |
| Diabetes mellitus | 12 (27) | 8 (40) |
| Congestive heart failure (NYHA 3–4) | 4 (9) | 1 (5) |
| COPD | 2 (4) | 1 (5) |
| Immunosuppressive condition | 4 (9) | 4 (20) |
| COVID-19 related admission | 28 (62) | 16 (80) |
| **Reason for ICU admission** |  |  |
| Shock | 5 (11) | 2 (10) |
| Acute respiratory failure | 33 (73) | 17 (85) |
| Coma | 4 (9) | 0 |
| Cardiac arrest | 3 (7) | 1 (5) |
| **Organ failures during ICU stay** |  |  |
| Vasopressors | 39 (87) | 20 (100) |
| Renal replacement therapy | 18 (40) | 11 (55) |
| Acute respiratory distress syndrome | 37 (82) | 18 (90) |
| Extracorporeal membrane oxygenation | 16 (36) | 7 (35) |
| Abbreviations: COPD, chronic obstructive pulmonary disease; ESBL-E, extended-spectrum β-lactamase-producing *Enterobacterales*; ICU, Intensive Care Unit; mPCR, multiplex polymerase chain reaction; NYHA, New York Heart association; SAPS, simplified acute physiology score; SOFA, sequential organ failure assessmentContinuous variables are expressed as median [interquartile range]; categorical variables are expressed as n (%).  ^1^Six patients had both one episode with and one without mPCR performed and are consequently included in the two groups | | |

| eTable 4. Results of the conventional microbiology testing for the 95 episodes of nosocomial pneumonia in the mechanically ventilated ESBL-E carriers | | | |
| --- | --- | --- | --- |
| **Variable** | **Conventional group, n=73** | **mPCR group, n=22** | **p-value** |
| Number of bacteria identified^1^ | 2 [1-2] | 1 [1-2] | 0.8 |
| Gram negative bacteria | 69 (95) | 20 (91) | 0.6 |
| *Enterobacterales* | 55 (75) | 13 (59) | 0.1 |
| *Escherichia coli* | 16 (22) | 3 (14) |  |
| *Klebsiella pneumoniae* | 22 (30) | 4 (18) |  |
| *Enterobacter cloacae* | 10 (14) | 7 (32) |  |
| *Klebsiella aerogenes* | 4 (5) | 0 |  |
| *Klebsiella oxytoca* | 2 (3) | 0 |  |
| *Proteus mirabillis* | 4 (5) | 0 |  |
| *Serratia marcescens* | 7 (10) | 0 |  |
| *Hafnia alvei* | 1 (1) | 0 |  |
| *Citrobacter koseri* | 1 (1) | 0 |  |
| *Raoultella ornithinolytica* | 0 | 1 (4) |  |
| **EBSL-E related pneumonia** | **38 (52)** | **9 (41)** | **0.4** |
| ***Escherichia coli*** | **14 (19)** | **1 (4)** |  |
| ***Klebsiella pneumoniae*** | **17 (23)** | **1 (4)** |  |
| ***Enterobacter cloacae*** | **7 (10)** | **7 (32)** |  |
| Nonfermenting gram-negative bacilli |  |  |  |
| *Pseudomonas aeruginosa* | 19 (26) | 9 (41) | 0.2 |
| *Stenotrophomonas maltophilia* | 11 (15) | 1 (4) | 0.3 |
| *Acineterobacter spp* | 5 (7) | 1 (4) |  |
| *Achromobacter xylosoxidans* | 5 (7) | 0 |  |
| *Burkholderia spp* | 2 (3) | 0 |  |
| *Haemophilus influenzae* | 0 | 1 (4) |  |
| *Prevotella spp* | 1 (1) | 0 |  |
| Gram positive bacteria | 11 (15) | 8 (36) | 0.07 |
| *Staphylococcus aureus* | 5 (7) | 5 (23) |  |
| *Streptococcus spp* | 3 (4) | 1(4) | 1 |
| *Corynebacterium striatum* | 4 (5) | 1 (4) |  |
| *Staphylococcus haemolyticus* | 0 | 1 (4) |  |
| Polymicrobial | 38 (52) | 11 (50) | 0.9 |
| Carbapenem resistant bacteria | 19 (26) | 5 (23) | 0.7 |
| *Pseudomonas aeruginosa^2^* | 4 (5) | 1 (4) |  |
| *Stenotrophomonas maltophilia* | 11 (15) | 1 (4) |  |
| Methicillin-resistant *Staphylococcus aureus* | 0 | 1 (4) |  |
| *Corynebacterium striatum* | 4 (5) | 1 (4) |  |
| *Staphylococcus haemolyticus* | 0 | 1 (4) |  |
| Abbreviations: ESBL-E, extended-spectrum β-lactamase-producing *Enterobacterales*  Categorical variables are expressed as n (%) and compared using Chi-square or Fisher’s exact tests as appropriate. No adjustment for multiple comparisons was performed.  ^1^The number of bacteria identified did not significantly differ according to sampling technique: protected telescope catheter (*N*=82), 2 [1-2]; bronchoalveolar lavage (*N*=7), 1 [1-2]; endotracheal aspirate (*N*=6), 1.5 [1-2], *p*=0.8 (Kruskal-Wallis rank sum test)  ^2^Carbapenem resistance was secondary to porin +/- efflux and/or mechanism (i.e., no carbapenemase was detected). | | | |

| **eTable 5. Factors associated with prescription of adequate empirical antibiotic therapy: results of univariate logistic regression analysis on the 95 episodes of nosocomial pneumonia in the mechanically ventilated ESBL-E carriers** | | | | |  |
| --- | --- | --- | --- | --- | --- |
| **Variable** | **Inactive empiric antibiotic therapy, n=45** | **Active empiric antibiotic therapy, n=50** | **Univariate OR  [95% CI]** | **p-value** | |
| Age, years | 49 [43-64] | 56 [43-65] | 1 [0.98-1.03] | 0.8 | |
| Women | 17 (38) | 21 (42) | 1.2 [0.52-2.7] | 0.7 | |
| Body mass index, kg/m² | 30 [26-33] | 30 [24-33] | 0.98 [0.92-1.05] | 0.6 | |
| **Comorbidities** |  |  |  |  | |
| Charlson Comorbidity index | 0 [0-1] | 0 [0-1] | 0.92 [0.7-1.21] | 0.6 | |
| Diabetes mellitus | 12 (27) | 14 (28) | 1.07 [0.4-2.6] | 0.9 | |
| COPD | 2 (4) | 1 (2) | 0.4 [0.04-5.1] | 0.6 | |
| Immunosuppressive condition | 5 (11) | 3 (6) | 0.5 [0.1-2.3] | 0.5 | |
| **Characteristics at ICU admission** |  |  |  |  | |
| COVID-19 related admission | 34 (76) | 37 (74) | 0.9 [0.4-2.3] | 0.9 | |
| SAPS II at ICU admission | 40 [27-60] | 43 [34-61] | 1.01 [0.99-1.04] | 0.2 | |
| **ESBL *Enterobacterales* colonisation** |  |  |  |  | |
| *Escherichia. Coli* alone | 20 (44) | 23 (46) | 1.06 [0.5-2.4] | 0.9 | |
| *Klebsiella Pneumoniae* and/or *Enterobacter Cloacae* | 21 (47) | 26 (52) | 1.2 [0.6-2.8] | 0.6 | |
| Others^1^ | 4 (9) | 1 (2) | 0.2 [0.02-1.9] | 0.2 | |
| **Patient characteristics at pneumonia onset** |  |  |  |  | |
| Days after ICU admission | 20 [11-50] | 23 [10-51] | 1 [1-1.01] | 0.2 | |
| Days after mechanical ventilation | 18 [11-50] | 19 [9-51] | 1 [1-1.01] | 0.2 | |
| Days after first positive ESBL-E carriage test | 11 [4-21] | 9 [3-37] | 1.01 [1-1.02] | 0.1 | |
| Previous VAP | 26 (58) | 30 (60) | 1.1 [0.5-2.5] | 0.8 | |
| Antibiotics received within the 72h prior to sampling | 25 (56) | 32 (64) | 1.4 [0.6-3.2] | 0.4 | |
| Carbapenem received within the 72h prior to sampling | 7 (16) | 10 (20) | 1.4 [0.5-3.9] | 0.6 | |
| Extracorporeal membrane oxygenation | 12 (27) | 16 (32) | 1.3 [0.5-3.1] | 0.6 | |
| SOFA score | 7 [4-9] | 7 [4-11] | 1.06 [0.95-1.2] | 0.3 | |
| PaO_2_/FiO_2_, mmHg | 182 [91-263] | 97 [71-165] | 1 [0.99-1] | 0.04 | |
| PaO_2_/FiO_2_ < 150 mmHg | 18 (40) | 31 (62) | 2.5 [1.1-5.7] | 0.03 | |
| Circulatory failure^2^ | 14 (31) | 31 (62) | 3.6 [1.6-8.7] | 0.003 | |
| Antibiotic therapy on the day of sampling | 15 (33) | 24 (48) | 1.8 [0.8-4.2] | 0.1 | |
| Non-carbapenem β-lactam | 13 (29) | 17 (34) | 1.3 [0.5-3] | 0.6 | |
| Carbapenem | 2 (4) | 7 (14) | 3.5 [0.7-17.9] | 0.2 | |
| **mPCR performed** | 3 (7) | 19 (38) | 8.6 [2.6-38.9] | 0.001 | |
| Abbreviations: ESBL-E, extended-spectrum β-lactamase-producing *Enterobacterales*; ICU, intensive care unit; mPCR, multiplex polymerase chain reaction; PaO_2_/FiO_2_, ratio of the partial pressure of arterial oxygen to the fraction of inspired oxygen; SOFA, sequential organ failure assessment; VAP, ventilator associated pneumonia; vHAP, ventilated hospital-acquired pneumonia  Continuous variables are expressed as median [interquartile range]; categorical variables are expressed as n (%)  ^1^*Citrobacter Koseri* (n=1), *Citrobacter Amalonaticus* (n=1), *Klebsiella Aerogenes* (n=1), *Klebsiella Oxytoca* (n=2)  ^2^Circulatory failure is defined as cardiovascular SOFA score of ≥ 3 | | | | |  |

| **eTable 6.** **Factors associated with prescription of adequate empirical antibiotic therapy among the 95 confirmed vHAP/VAP** | | | | | | | | |
| --- | --- | --- | --- | --- | --- | --- | --- | --- |
| **Variable** | **Crude* *N*= 95** | | **Multivariable analysis† *N*= 95** | | **Propensity-weighting cohort‡ *N* = 95** | | **Matching-cohort *N* = 44** | |
|  | OR [95% CI] | p value | ORa [95% CI] | p value | aOR [95% CI] | p value | aOR [95% CI] | p value |
| **mPCR performed** | | | | | | | | |
| No | 1 |  | 1 |  | 1 |  |  |  |
| **Yes** | **8.6 [2.6-38.9]** | **0.001** | **7.5 [2.1-35.9]** | **0.004** | **5.9 [1.6-22.1]** | **0.008** | **5.8 [1.5-22.1]** | **0.01** |
| Circulatory failure^1^ | | | | | | | | |
| No | 1 |  | 1 |  |  |  |  |  |
| Yes | 3.6 [1.6-8.7] | 0.003 | 3.1 [1.2-8.2] | 0.02 |  |  |  |  |
| PaO_2_/FiO_2_ < 150 mmHg | | | | | | | | |
| No | 1 |  | 1 |  |  |  |  |  |
| Yes | 2.5 [1.1-5.7] | 0.03 | 2.2 [0.9-5.9] | 0.1 |  |  |  |  |
| Carbapenem received within 72h prior to sample | | | | | | | | |
| No | 1 |  | 1 |  |  |  |  |  |
| Yes | 1.4 [0.5-3.9] | 0.6 | 2.0 [0.6-6.7] | 0.2 |  |  |  |  |
| Abbreviations: ESBL-E, extended-spectrum β-lactamase-producing *Enterobacterales*; ICU, intensive care unit; mPCR, multiplex polymerase chain reaction; OR [95% CI] odds ratio [95% confidence interval]; PaO_2_/FiO_2_, ratio of the partial pressure of arterial oxygen to the fraction of inspired oxygen; SOFA, sequential organ failure assessment; VAP, ventilator associated pneumonia; vHAP, ventilated hospital-acquired pneumonia  ^1^Circulatory failure was defined as cardiovascular SOFA score of ≥ 3  *Univariable logistic regression  †Multivariable logistic regression models, adjusting for the use of mPCR, circulatory failure, PaO2/FiO2 < 150 mmHg, and the use of carbapenem within the 72 hours prior sampling. The multivariable model showed a good calibration as assessed by the Hosmer and Lemeshow goodness-of-fit test (p = 0.9603) and a fair discrimination as assessed by the receiver operating characteristics curve (area under the curve = 0.7853).  ‡Propensity-score weighted analysis using overlap weighting, with the use of mPCR as the dependent variable and the following characteristics at pneumonia onset as covariates: circulatory failure defined as cardiovascular SOFA score of ≥ 3, SOFA score and the use of carbapenem within the 72 hours prior to sampling | | | | | | | | |

| **eTable 7. Factors associated with the prescription of adequate empirical antibiotic therapy in mechanically ventilated ESBL-E carriers (*N*=59) with first episode of nosocomial pneumonia.** | | | | | | | | |
| --- | --- | --- | --- | --- | --- | --- | --- | --- |
| **Variable** | **Crude* *N*= 59** | | **Multivariable analysis† *N*= 59** | | **Propensity-weighting cohort‡ *N* = 59** | | **Matching-cohort *N* = 34** | |
|  | OR [95% CI] | p value | ORa [95% CI] | p value | aOR [95% CI] | p value | aOR [95% CI] | p value |
| **mPCR performed** | | | | | | | | |
| No | 1 |  | 1 |  | 1 |  |  |  |
| **Yes** | **11 [2.7-76]** | **0.003** | **10.3 [2.1-80.1]** | **0.009** | **8.1 [1.8-36.4]** | **0.006** | **7 [1.5-33.1]** | **0.01** |
| Circulatory failure^1^ | | | | | | | | |
| No | 1 |  | 1 |  |  |  |  |  |
| Yes | 5.2 [1.8-16.7] | 0.004 | 6.9 [2-29] | 0.004 |  |  |  |  |
| PaO_2_/FiO_2_ < 150 mmHg | | | | | | | | |
| No | 1 |  | 1 |  |  |  |  |  |
| Yes | 2.8 [1-8.4] | 0.05 | 2.5 [0.7-10] | 0.2 |  |  |  |  |
| Carbapenem received within 72h prior to sample | | | | | | | | |
| No | 1 |  | 1 |  |  |  |  |  |
| Yes | 0.8 [0.1-7.3] | 0.9 | 0.9 [0.1-9] | 0.9 |  |  |  |  |
| Abbreviations: ESBL-E, extended-spectrum β-lactamase-producing *Enterobacterales;* ICU, intensive care unit; mPCR, multiplex polymerase chain reaction; OR [95% CI] odds ratio [95% confidence interval]; PaO_2_/FiO_2_, ratio of the partial pressure of arterial oxygen to the fraction of inspired oxygen; SOFA, sequential organ failure assessment.  ^1^Circulatory failure is defined as cardiovascular SOFA score of ≥ 3  *Univariable logistic regression  †Multivariable logistic regression model, adjusting for the use of mPCR, circulatory failure, PaO2/FiO2 of < 150 mmHg, and the use of carbapenem within the 72 hours prior to sampling. The multivariable model showed a good calibration as assessed by the Hosmer and Lemeshow goodness-of-fit test (p = 0.8139) and a fair discrimination as assessed by the receiver operating characteristics curve (area under the curve = 0.8322).  ‡Propensity-score weighting analysis using overlap weighting, with the use of mPCR as the dependent variable and the following characteristics at pneumonia onset as covariates: circulatory failure defined as cardiovascular SOFA score of ≥ 3, PaO2/FiO2 of < 150 mmHg, and the use of carbapenem within the 72 hours prior to sampling. | | | | | | | | |
